# One View of Heredity

**Published:** 1893-06

**Authors:** Augustin Prichard

**Affiliations:** Consulting Surgeon to the Bristol Royal Infirmary


### ONE VIEW OF HEREDITY.

BY

AUGUSTIN PRICHARD, F.R.C.S. Eng., M.D. Berlin,
*Consulting Surgeon to the Bristol Royal Infirmary.*

---

IN one of the very many casual and pleasant little conversations I have had with a very good medical friend, of high standing, and hailing, I imagine, from some district north of Bristol, we were talking of the well-known effect of heredity in the moral as well as in the physical constitution of man, a fact generally acknowledged by the use of such expressions as "A chip of the old block," &c.; and he propounded, perhaps not quite seriously, the doctrine that the right treatment and punishment for the habitual criminal (by which is understood that numerous class of men who spend their lives in prison, being committed again and again for the same offence, after having been free probably for only a few days) would be the operation of castration. This would, at any rate, interfere with the continuation of the vicious race, as far as that individual was concerned; and I agreed with his idea. But where did he get the notion? Where could we find a Government strong enough to propose such a remedy? The very existence of such a law among our statutes would of itself deter many from crime. Is it a practical or practicable suggestion? That it would sensibly and materially lessen the number of the criminal population, there can be no doubt whatever; and it would begin at the right end of the difficulty by stopping the supply of children who, from their surroundings, and from the inevitable inherited taint in their blood, would almost of necessity grow up to follow the vicious steps of their parents; and no microscope will ever find the germ of this disease.

I have in my possession a little parchment-bound duodecimo volume, being an account of Scotland, Ireland, and Scandinavia, 1627: "*Scotiæ, Hiberniæ et Sveciæ Descriptio;* Lugd. Bat. CIƆ IƆ CXXVII." The Irish people in early times, according to this writer, sufficiently resembled the present inhabitants of the country to be a convincing proof of heredity after all these centuries. With reference to the climate, the author says that it is so damp that strangers, when they first come, are subject to diarrhœa and dysentery, "for the cure of which, however, they have a certain wholesome *aqua vitæ* (uskebach [1] *vocãt*);" and the inhabitants are divided into two parts; namely, those who "refuse to obey the laws, and live in an uncivilised way (*vulgo* VVilde Irish, *id est*, *sylvestres Hibernici*, *vocantur*), and others who recognise the power of the laws, and live in a civilised way on the eastern and more fertile part of the country (The English Pale)."

But it is principally on account of the description of the inhabitants of Scotland and their customs, as given by this old writer, whose book seems to be chiefly a compilation from other and older sources, that I have referred to this subject. He speaks of the bravery of the Scotch, of their fondness for variegated and striped coloured cloaks, and says: "*In victu, vestitu, totaque rei domesticæ ratione, antiqua utuntur parsimonia.*" He describes their love of sport, but he alludes to the Highlanders of old times as a nation of savages:

"When going out to battle they dipped the points of their swords in the blood of the first animal they met, and tasted it, and this was considered a pledge of prosperity. . . . In carrying on their wars, as well as in their private quarrels, they did nothing by deceit or fraud, thinking it noble to fight and conquer in open battle; and they considered it base to conceal their enmity by flattering speeches and afterwards to attack those who were unprepared . . . ." And then comes the following statement, which induced me to give an account of this old book in these pages, which are generally occupied with scientific and useful work: "Men who had been found after much inquiry to be suffering from epilepsy, idiocy, mania, or any similar disease, which could easily be transmitted to their offspring, were castrated, that the race should not be contaminated by foul contagion by those who might be born to them;

[1] An old Irish word, meaning "water of life," the original of the word "whisky."

and women who were found to be infected with any of these diseases, or with leprosy, were kept far away from any association with men; but if one of these be found to have conceived, she was buried alive with her unborn child; and, to prevent disgrace to their country, gluttons, unnatural feeders and gormandisers, and habitual drunkards were punished in a mild way (*miti supplicio exterminarunt*), being first allowed to eat and drink as much as they liked, and then drowned in the river."

I cannot fairly suggest that a far-fetched heredity induced my friend to make his remark on the treatment of habitual criminals any more than I can ask credit for all that is told us of the barbarous habits of his remote ancestors.

